# Detection of Very Long Antisense Transcripts by Whole Transcriptome RNA-Seq Analysis of *Listeria monocytogenes* by Semiconductor Sequencing Technology

**DOI:** 10.1371/journal.pone.0108639

**Published:** 2014-10-06

**Authors:** Stefanie Wehner, Gopala K. Mannala, Xiaoxing Qing, Ramakanth Madhugiri, Trinad Chakraborty, Mobarak A. Mraheil, Torsten Hain, Manja Marz

**Affiliations:** 1 Faculty of Mathematics and Computer Science, Friedrich-Schiller-University Jena, Jena, Germany; 2 Institute of Medical Microbiology, Justus-Liebig-University Giessen, Giessen, Germany; 3 German Center for Infection Research (DZIF), Partner site Giessen-Marburg-Langen, Giessen, Germany; 4 Institute of Medical Virology, Justus-Liebig-University Giessen, Giessen, Germany; University of Illinois at Chicago College of Medicine, United States of America

## Abstract

The Gram-positive bacterium *Listeria monocytogenes* is the causative agent of listeriosis, a severe food-borne infection characterised by abortion, septicaemia, or meningoencephalitis. *L. monocytogenes* causes outbreaks of febrile gastroenteritis and accounts for community-acquired bacterial meningitis in humans. Listeriosis has one of the highest mortality rates (up to 30%) of all food-borne infections. This human pathogenic bacterium is an important model organism for biomedical research to investigate cell-mediated immunity. *L. monocytogenes* is also one of the best characterised bacterial systems for the molecular analysis of intracellular parasitism. Recently several transcriptomic studies have also made the ubiquitous distributed bacterium as a model to understand mechanisms of gene regulation from the environment to the infected host on the level of mRNA and non-coding RNAs (ncRNAs). We have used semiconductor sequencing technology for RNA-seq to investigate the repertoire of listerial ncRNAs under extra- and intracellular growth conditions. Furthermore, we applied a new bioinformatic analysis pipeline for detection, comparative genomics and structural conservation to identify ncRNAs. With this work, in total, 741 ncRNA locations of potential ncRNA candidates are now known for *L. monocytogenes*, of which 611 ncRNA candidates were identified by RNA-seq. 441 transcribed ncRNAs have never been described before. Among these, we identified novel long non-coding antisense RNAs with a length of up to 5,400 nt e.g. opposite to genes coding for internalins, methylases or a high-affinity potassium uptake system, namely the *kdpABC* operon, which were confirmed by qRT-PCR analysis. RNA-seq, comparative genomics and structural conservation of *L. monocytogenes* ncRNAs illustrate that this human pathogen uses a large number and repertoire of ncRNA including novel long antisense RNAs, which could be important for intracellular survival within the infected eukaryotic host.

## Introduction

*Listeria monocytogenes* is a non-sporulating, Gram-positive soil bacterium which has a low GC content. The ubiquitous nature of the bacterium enables it to enter the human food chain via food-processing environments. In addition, the ability of *L. monocytogenes* to grow at low temperatures and to resist harsh preservation techniques increases the risk of food contamination. By uptake via contaminated food products, *L. monocytogenes* can cause listerial infection known as listeriosis. Listeriosis often manifests with clinical symptoms such as meningitis, meningoencephalitis, septicaemia, abortion, prenatal infection and also gastroenteritis. Furthermore, high mortality rates of up to 20–30% in humans which are diseased with listeriosis (especially pregnant women, elderly and immunocompromised persons) makes *L. monocytogenes* a serious life-threatening human pathogen [1], [2].

The genus *Listeria* consists of ten species, *L. monocytogenes, L. ivanovii, L. seeligeri, L. innocua, L. marthii, L. welshimeri, L. rocourtiae, L. weihenstephanensis, L. grayi* and *L. fleischmannii. L. monocytogenes* and *L. ivanovii* are the only known pathogens of this group [3]–[8].

Comparative whole genome sequencing of representative strains comprising the entire species of *L. monocytogenes* was performed by Kuenne *et al.* [9]. In the genus *Listeria*, genome reduction has led to the generation of non-pathogenic species from pathogenic progenitor strains [10]. Indeed, many of the genomic regions specific for pathogenic species (such as *L. monocytogenes*) represent genes which are absent in non-pathogenic species (such as *L. innocua* and *L. welshimeri*) [10]. This also effects the number of conserved non-coding RNAs (ncRNAs) within the genus *Listeria* [9], [11]. Recently genome sequencing of different *L. monocytogenes* serotypes has been accompanied by transcriptional profiling using whole genome microarrays and RNA-seq. This has been done to examine the adaptive changes of *L. monocytogenes* to grow in different natural environments and to identify responsible genes and ncRNAs mediating transcriptional responses [9], [11]–[15]. For *L. monocytogenes*, 262 ncRNAs have been identified yet including 134 putative sRNAs, 86 antisense RNAs (asRNAs) and 42 riboswitches [16]. Also in other bacteria, asRNA transcripts could be observed for 10% up to 50% of protein-coding genes, e.g. in *Escherichia coli*, *Synechocystis* sp. PCC6803, *Helicobacter pylori* [17], *Bacillus subtilis* [18] and *Mycobaterium tuberculosis* [19].

In this study we present information on transcriptomic profiling using RNA-seq, comparative genomics and structural conservation of *L. monocytogenes* ncRNAs. The bacterial strains have been grown in BHI broth (extracellular conditions) and in the cytosolic environment of the host cell (intracellular condition). To our best knowledge, this is the first time that Ion Torrents Personal Genome Machine (PGM) (Life Technologies) was used for RNA-seq analysis of a bacterial human pathogen by next generation semiconductor sequencing technology to detect novel small and long ncRNAs. Using this technology, we found antisense transcripts in *Listeria* with a length up to 5,400 nt.

## Materials and Methods

### Bacterial strains and growth conditions

The strains *L. monocytogenes* EGD-e [20], *L. monocytogenes* 1043S [21] and *L. monocytogenes* EGD-e Δ*prfA* [22] were grown in BHI broth (VWR) overnight at 37°C with shaking at 180 rpm (Unitron, Infors). Overnight cultures were diluted 1∶50 in 20 ml fresh BHI broth using a 100 ml Erlenmeyer flask and were incubated at the same conditions mentioned above until OD_600 *nm*_ 1.0.

### Cell culture and infection model

P388D1 murine macrophage cells (ATCC CCL-46) were cultured in RPMI1640 (Gibco) supplemented with 10% fetal calf serum (PAA Laboratories) in 85-mm-diameter tissue culture plates. For intracellular growth assays bacteria were added to P388D1 murine macrophages monolayer at a multiplicity of infection (MOI) of 10 *Listeria* per eukaryotic cell. The intracellular growth assays were performed as described in [23].

### RNA isolation

For RNA extraction from *L. monocytogenes* grown extracellularly in BHI, we applied aliquots of 0.5 ml from the same *Listeria* culture grown until mid-exponential phase used to infect P388D1 macrophages. The bacterial cells were treated with 1.0 ml RNA protect (Qiagen) for 5 min and were collected by centrifugation for 10 min (8000 g). The bacterial pellets were stored at −80°C until use. RNA extraction from intracellularly grown *L. monocytogenes* in macrophages, 4 h post infection, was performed as described previously [23]. Briefly, infected host cells (see above: Cell culture and infection model part) were lysed using cold mix of 0.1% (wt/vol) sodium dodecyl sulfate, 1.0% (vol/vol) acidic phenol and 19% (vol/vol) ethanol in water. The bacterial pellets were collected by centrifugation for 3 min (16,000 g). Total RNA was extracted using miRNeasy kit (Qiagen) with some modifications [11]. The collected pellets were washed with SET buffer (50 mM NaCl, 5 mM EDTA and 30 mM Tris-HCl (pH 7.0)). After centrifugation at 16000 g for 3 min pellets were resuspended into 0.1 ml Tris-HCl (pH 6.5) containing 50 mg/ml lysozyme (Sigma), 25 U of mutanolysin (Sigma), 40 U of SUPERase (Ambion), 0.2 mg of proteinase K (Ambion). The incubation for 30 min was carried out on a thermo mixer at 37°C and with shaking (350 rpm). QIAzol (Qiagen) was added, mixed gently and incubated for 3 min at room temperature. An additional incubation for 2 min at room temperature was done after adding 0.2 volume chloroform followed by centrifugation at 16000 g at 4°C for 15 min. The upper aqueous phase, containing RNA, was transferred to a new collection tube and 1.5 volumes of 100% ethanol was added and mixed thoroughly. The probes containing RNA were transferred into columns supplied with the miRNeasy Kit (Qiagen) and treated according to the manual including an on-column DNase digestion (RNase-Free DNase, Qiagen). RNA was eluted by RNase-free water and stored at −80°C until needed. The quantity of the isolated total RNA was determined by absorbance at 260 nm and 280 nm, and the quality was assessed using Nano-chips for Agilents 2100 Bioanalyzer.

### RNA sequencing

To deplete bacterial rRNA we applied the Ribo-Zero Magnetic Kit (Bacteria) (Epicentre) and treated the depleted RNA with tobaco acid pyrophosphatase (Epicentre) as recommended by the manufacturer.

Afterwards, the RNA was fragmented by RNase III (Applied Biosystems) at 37°C for 4 min. The yield and size distribution of the fragmented RNA was assessed using Quant-iT RNA assay kit with Qubit Fluorometer (Invitrogen) and the Agilent RNA 6000 Pico Chip kit with Agilent 2100 Bioanalyzer instrument. Size distribution of RNase III fragmented RNA delivered median size of 200 nt. For the cDNA library preparation, Ion Total RNA-seq kit v2 (Ion Torrent, Life Technologies) was used as recommended by the manufacturer. The libraries were purified by AMPure XP Reagent (Beckman Coulter). The yield and size distribution of the amplified cDNA were assessed by Qubit Fluorometer (Invitrogen) and DNA 1000 kit (Agilent). In the next step, clonally amplified Ion Sphere Particles (ISPs) containing the amplified cDNA were prepared using the Ion OneTouch System (Life Technologies). The amplified libraries were diluted to 8.3 nM and loaded on 316 Chip of the Ion Torrent semiconductor sequencing instrument personal genome machine (PGM) (Life Technologies).

### Real-time-RT-PCR

Reverse transcription to produce cDNA was performed by SuperScript II Reverse Transcriptase (Invitrogen) using 1 *μ*g RNA. The probes were subjected to quantitative real-time PCR in a final volume of 25 *μ*l using QuantiTect SYBR Green PCR kit (Qiagen) according to the manufacturers instruction. A standard curve was generated for the used primer pairs (see supplemental material) using different copy numbers of genomic DNA from *L. monocytogenes* EGD-e. For each primer pair a negative control (water), RNA sample without reverse transcriptase (to determine genomic DNA contamination) and a sample with known amount of copy numbers (to test the efficiency of the reaction) were included as controls during cDNA quantification. After real-time PCR all samples were run on a 1.5% agarose gel to verify that only a single band was produced. The expression level of each gene was calculated by normalizing its mRNA quantity to the quantity of the mRNA of *gyrB* encoding gyrase B [24] for the same sample using a mathematical model for relative quantification in real-time PCR published by Pfaffl [25].

### *In silico* Genome Data Analysis

In order to analyze the genome of *L. monocytogenes* (NC_003210) with RNA-seq data and to detect potential novel ncRNAs, we investigated the genome searching for: (a) proteins, (b) known ncRNAs, (c) conserved regions, (d) locally stable structures, (e) possible *de novo* ncRNAs, and (f) positions of known potential small RNAs from literature [13], [15], [26].

#### Annotation of known proteins

Protein annotation from NCBI (NC_003210) was extended by a *de novo* protein prediction with BacProt [27] based on homologous proteins of other firmicutes. Furthermore, BacProt predicts species specific novel proteins based on *Listeria* specific information on Shine-Dalgarno sequences and TATA boxes gained from the homology search.

#### Annotation of known ncRNAs

tRNAs were annotated using tRNAscan-SE (v.1.23) [28] with parameters -omlfrF. For the annotation of ribosomal RNAs (rRNAs), we used rnammer (v.1.2) [29] with the parameters -S bac -m lsu,ssu,tsu.

For the other ncRNA classes, homology searches using BLAST (v.2.2.21) [30] (E-Value: E<10^−4^) and infernal (v.1.0.2) [31] were performed. Known sequences of the corresponding classes, which were downloaded from Rfam database (v.10.0) [32], were used as input.

#### Conserved regions: multiple genome-wide alignfment

The multiple genome-wide alignment was calculated using POMAGO [33] with *L. monocytogenes* EGD-e as reference species. The following organisms were included into the multiple genome-wide alignment analysis: *L. monocytogenes* ATCC 19117, *L. monocytogenes* CLIP80459, *L. monocytogenes* FSL J1-208, *L. monocytogenes* L99, *L. monocytogenes* SLCC2482, *L. monocytogenes* SLCC2372, *L. monocytogenes* SLCC2376, *L. monocytogenes* SLCC2378, *L. monocytogenes* SLCC2479, *L. monocytogenes* SLCC2540, *L. monocytogenes* SLCC2755 and *L. monocytogenes* SLCC7179.

#### Annotation of *de novo* ncRNAs via RNAz

Based on the calculated multiple genome-wide alignment an RNAz-analysis --cutoff  = 0.5 (v.2.1) [34] was performed.

#### Locally stable secondary structures

Locally stable secondary structures are indicating positions for small RNAs. Those structures were calculated with RNALfold (v.2.0.7) [35] using parameters -d 2 -L 120. Hits with a total length less than 50 nt were discarded. A dinucluotide shuffling of each sequence with shuffle -d -n 1000 was performed to predict thermodynamically stable RNA structures. For further analyses only extraordinarily stable structures with a Z-score cut-off ≤−3.0 (top 5% of stable structures) were taken into account.

### Transcriptome data analysis

Reads were clipped with fastx-clipper (v. 0.0.13) (http://hannonlab.cshl.edu/fastx_toolkit/). All reads from one growth condition were merged to one library and then mapped to the *L. monocytogenes* EGD-e genome (NC_003210) by segemehl (v.0.1.3–335) [36] using standard paramaters (-A 85 -e 5). For normalisation the number of all mapped reads (except rRNAs and tRNAs) of the two libraries were used.

#### Detection of possible *de novo* non-coding RNAs

For the detection of potential novel non-coding RNAs, all intergenic regions with a minimum length of 10 nt and a minimum coverage of ten reads were defined as ‘seeds’. For the analysis of long (antisense) non-coding RNAs, we merged seed regions, with a distance less than 100 nt. All candidates were scored according to the characteristics of known ncRNAs of Rfam [37] and from previously identified ncRNAs [13], [15], [26] to indicate possible novel ncRNAs (Tab. 1, supplemental material (http://www.rna.uni-jena.de/supplements/listeria/).

**Table 1:** Scoring system.

Criterion	Score					
Length (nt)	>50	+0.25	>75	+0.25	>100	+0.5
Reads	>9	+1	>100	+1		
GC (%)	>40	+0.25	>50	+0.25		
RNALfold		+0.25				
POMAGO	= 13	+0.25				
RNAz (p)	>0.9	+0.25				

For evaluation of the ncRNA candidates, a scoring system retrieved from known ncRNAs (Rfam, [13], [15], [26], see supplemental material) was developed. For increasing length, number of reads and GC content, scores are summed up along the column; for example, an ncRNA candidate of length 100 nt receives a score of +1. The higher the score of a candidate, the higher its probability to be an ncRNA.

For further analyses, we took only candidates with a score of 2.5 or higher into account. Additionally, we checked our candidates for possible overlaps with the 5′UTR predicted by Wurtzel *et al.* [15].

## Results and Discussion

### Full ncRNA candidate set

In this study we analyzed the transciptomes of *L. monocytogenes* grown extracellularly in BHI broth and *L. monocytogenes* grown intracellularly in murine macrophages. Our analysis was based on three independent biological replicates for each condition resulting in six RNA-seq libraries produced by the Ion torrent (PGM) next generation sequencing platform. We obtained 3.1–3.7 million reads up to a length of 385 nt (see Tab. 2).

**Table 2:** Overview of RNA-seq libraries.

Library	Number of reads		Read length	Mean read length	
	before clipping	after clipping		before clipping	after clipping
intra-1	3,253,920	3,151,751	6–368	106.613	85.7815
intra-2	3,412,934	3,322,309	8–374	156.797	116.062
intra-3	3,748,637	3,660,315	8–385	150.629	107.838
extra-1	3,165,988	3,079,495	6–365	108.007	82.53
extra-2	3,322,796	3,247,113	6–371	138.98	102.825
extra-3	3,710,603	3,660,845	6–362	157.823	114.506

Libraries were retrieved by next generation semiconductor sequencing technology. Number of reads before and after clipping and their mean length.

The experimental approach was combined with comprehensive *in silico* studies. To detect novel ncRNAs, we investigated various characteristic features of ncRNAs in the *L. monocytogenes* genome and transcriptome: seeds, GC-content, secondary structure, conservation and multiple genome-wide alignment.

1. A *seed* is defined by an intergenic region covered by ≥10 reads for ≥10 nt. We searched for seeds and merged them to one candidate if they were at most 100 nt apart. We received 2074 candidate ncRNA locations. Locations longer than 50 nt, 75 nt and 100 nt were rewarded by +0.25, +0.5 and +1 respectively (see Tab. 1). If the number of reads was at least ten, the score of the ncRNA candidate was increased by 1. If the number of reads even exceeded 100, the score was again increased by 1.
2. We analyzed the GC-content. The whole genome of *L. monocytogenes* EGD-e has an GC content of 38%. The ncRNAs of Rfam identified in *L. monocytogenes* EGD-e were found to have an GC content of 52% and 44% (with and without rRNAs/tRNAs). We decided to reward ncRNAs with GC content above 40% with 0.25, and another 0.25 points for GC content above 50%. However, previously reported ncRNAs [13], [15], [26] showed a lower GC content (on average 37%, 37.8% and 37.6% respectively).
3. Using RNALfold we searched for locally stable secondary structures. For 87/143 ncRNAs described in Rfam and 118/260 ncRNA candidates previously described in the literature [13], [15], [26], we found a region which was identified by RNALfold as locally stable secondary structure. If a candidate was predicted to contain a locally stable secondary structure region, we rewarded this candidate by adding +0.25 to its score.
4. Another hint for an (ncRNA) gene is its conservation among closely related species. Therefore, we computed a genome-wide multiple sequence alignment comparing *L. monocytogenes* EGD-e with 12 other *L. monocytogenes* serotypes. If the candidate region was present in all other serotypes, the candidate was rewarded by adding another +0.25 to its score.
5. The multiple genome-wide alignment was used as input for RNAz to predict novel ncRNAs. If a candidate was identified to be a novel ncRNA with probability above 0.9, we added another +0.25 to its score.

For the further analysis we took only those novel ncRNA candidates into account that exceeded a given threshold. We chose this threshold by checking how many of the previously described ncRNAs would have been selected. For a threshold of 2.5, 132/143 of the ncRNAs described in Rfam and 137/260 of the previously putative ncRNAs described in the literature, would have been selected. Using this threshold, we present a set of 441 potential novel ncRNA candidates. To get a full set of ncRNA locations, we added the previously described ncRNAs to our set of novel ncRNA candidates. This results in 741 ncRNA locations (since both sets are overlapping), ranging from to 10–5,347 nt (mean: 239 nt) length for *L. monocytogenes*. If we use our threshold also for the previously described ncRNA locations, we get a set of 611 ncRNA candidates. The list of all candidates, their genomic locations and features as described above, as well as overlaps to previously described ncRNAs and adjacent proteins is given in the supplemental material.

### Comparison to previous studies

**Figure 1:**
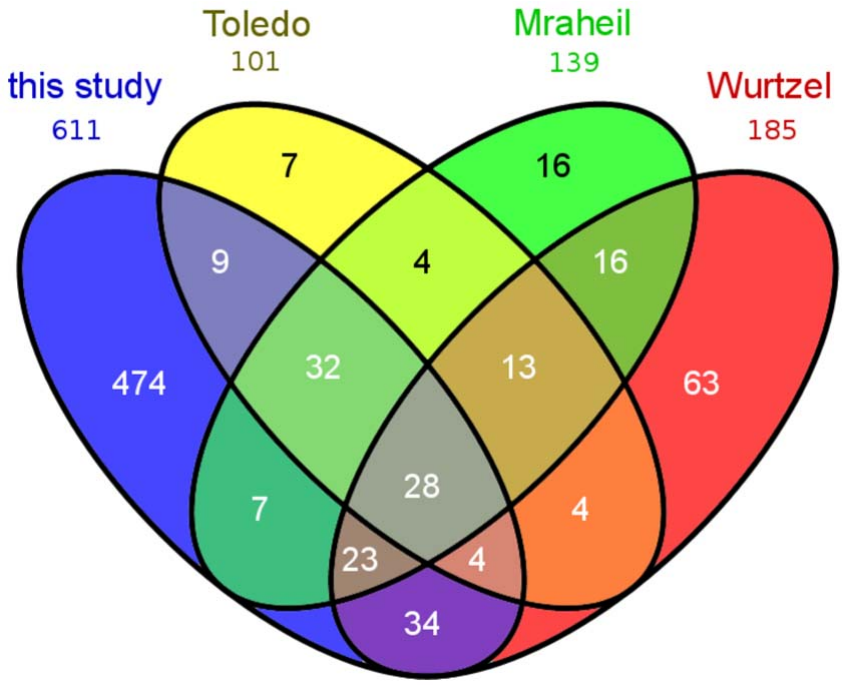
Comparative analysis of ncRNA transcriptome data: Comparison of our ncRNA candidates with results of previous studies performed by Toledo-Arana *et al.* [13], Mraheil *et al.* [11] and Wurtzel *et al.* [15]. Note that whenever an ncRNA prediction of this study overlaps with multiple previously described candidates, it is a single hit in the diagram. Altogether, including previous literature, Rfam and this work, now 741 putative ncRNAs are described. In this work we defined 611 to be putative ncRNAs, of which 474 ncRNAs are not part of previous literature, 33 of them known ncRNAs from Rfam.

As mentioned above, 260 locations of ncRNA candidates (including start- and stop positions) were previously described in the literature [11], [13], [15]. We compared our 611 ncRNA candidates with the results of these previous studies (see Fig. 1).

In 2009, Toledo-Arana *et al.* [13] used tiling arrays and RNAs from wild type and mutants grown *in vitro*, *ex vivo*, and *in vivo*, to present a complete operon map of *L. monocytogenes*. In this study, 100 ncRNA candidates were suggested. Of this 100 putative sRNAs, 77 locations were also confirmed by our observations, whereas 23 locations had a score ≤2.5 or were not even identified as seeds.

Mraheil *et al.* [11] reported 150 putative regulatory RNAs identified by deep sequencing with cDNA obtained from extracellularly grown bacteria and from *L. monocytogenes* isolated from infected macrophages using 454 pyrosequencing. From these 150 putative regulatory RNAs, we identified 102 using our method and a score threshold of 2.5. More than half of the remaining 48 ncRNAs were covered with less than 10 reads and were not part of our seeds.

Wurtzel *et al.* [15] performed a comparative study of *L. monocytogenes* and the non-pathogenic *L. innocua* using strand-specific cDNA sequencing. This resulted in genome-wide transcription start site maps and the identification of 183 ncRNAs. From the 183 reported ncRNAs, 100 were identified by our method, whereas half of the remaining ncRNAs were lacking expression.

Interestingly, there were a few examples where Wurtzel *et al.* [15] described a long candidate, which was covered by two or more candidates from our putative ncRNA set. These regions were discovered as several candidates by our method, since the expression pattern dropped down in between the candidates. The most noticeable example is anti1846 with a described length of 1371 nt, which overlaps with four of our candidates (216 nt, 141 nt, 23 nt and 227 nt).

In general, our method rather predicted longer ncRNAs which overlap with two or more previously described ncRNAs. For example, *LhrC-1*–*LhrC-4* were reported earlier as four ncRNA candidates [15] and have been merged by our approach to a single putative ncRNA, which conforms to the first description of this ncRNA by Christiansen *et al.* [38] in 2006. But even though the complete region was covered, the expression was not continuously on the same level.

Nevertheless, we missed a few of the ncRNA candidates described in previous studies (see Fig. 1). This can be attributed to the differences in the experimental setup: we used a different sequencing technology, different organisms at different expression time points, and a different subsequent *in silico* scoring. From the previously reported ncRNA candidates that were actually covered by reads, only a small fraction was rejected by our filtering steps.

From the 611 ncRNAs detected by our method, 474 were identified here by RNA-seq for the first time. From these, 33 candidates were already known from Rfam and 441 have, as far as we know, never been reported before.

**Figure 2:**
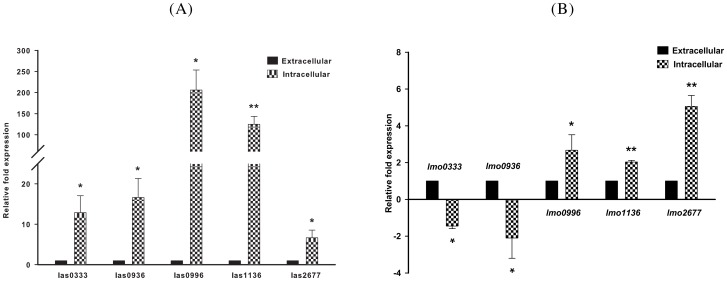
Validation of new long antisense (las) RNAs in *L. monocytogenes* by qRT-PCR analysis. (A) The presence of las transcripts was determined by strand-specific qRT-PCR analysis. Supporting the results of RNA-seq, the qRT-PCR analysis indicated that the novel lasRNA transcripts las0333, las0936, las0996, las1136 and las2677 were significantly up-regulated in intracellular conditions. ‘*’ −*P*≤0.05 ‘**’ −*P*≤0.01. (B) Strand specific qRT-PCR analysis of las respective target genes shows significant downregulation of *lmo0333* (internalin), and *lmo0936* (nitroflavin reductase), upregulation of *lmo0996* (methyltransferase), *lmo1136* (internalin) and *lmo2677* (esterase) in intracellular growth condtions. ‘*’ −*P*≤0.05; ‘**’ −*P*≤0.01. Primers used for qRT-PCR are available at the online Supplemental Material.

In our set of predicted ncRNAs we found some highly interesting (long-)antisense ncRNAs (lasRNAs) with up to 5,400 nt, which were induced under intracellular conditions. Most of the lasRNAs described below were validated by qRT-PCR (Fig. 2).

### Internalins are very likely controlled by our detected lasRNAs

**Figure 3:**
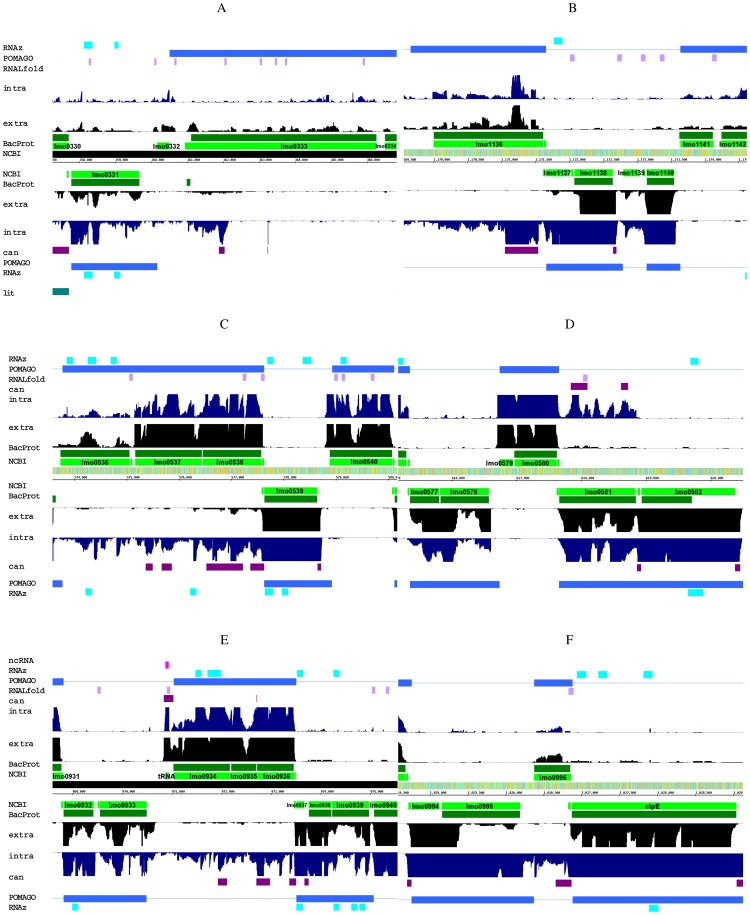
Transcription of selected long asRNAs (lasRNAs): (A) Internalin protein; (B) Internalin protein (note the different scales of x-axis); (C) a novel long antisense transcript with more than 2,400–3,800 nt; (D) predicted SAM-dependent methyltransferase; (E) a rRNA methylase homolog; (F) similar to a methylated DNA protein cystein methyltransferase (note the different scales of x-axis). The upper half of each transcription profile represents the plus strand and the lower one the minus strand. Number of displayed reads is limited to 20. Dark purple – detected ncRNA candidates; lightgreen – NCBI annotation; darkgreen – BacProt annotation; black – reads of the extracellular library; dark blue – reads of the intracellular library; violet – locally stable secondary structure (analyzed with RNALfold); blue – conserved region among other *L. monocytogenes* serotypes (analyzed with POMAGO); cyan blue – potential new ncRNAs predicted by RNAz; pink – annotated ncRNAs. A better resolution of the figure can be found in the supplement.

Two long ncRNA candidates were detected as antisense transcripts of two genes coding for the proteins *lmo0333* and *lmo1136* (see Tab. 3, and Fig. 3A,B). Both proteins *lmo0333* and *lmo1136* are similar to internalin proteins (according to NCBI annotation) and contain an LRR-LPXTG-motif.

**Table 3:** Selected candidates.

Fig.	UpOrf		DownOrf		Start	Stop	S	cL(Size)	GC	IC	EC	P	RNAz	RNAL	Antisense	Score
Antisense transcript of proteins with LRR+LPXTG motif																
3A	lmo0333	+	lmo0334	+	361885	362047	−	163(163)	0.410	28	0	13	.	−18.80	lmo0333	3
3A	lmo0333	+	lmo0334	+	363242	363256	−	15(15)	0.420	121	0	13	.	.	lmo0333	2.5
3B	lmo1136	+	lmo1137	−	1171054	1171546	−	493(493)	0.449	87	0	13	.	.	lmo1136	2.75
Novel long antisense transcript (2,400 nt–3,800 nt)																
3C	lmo0537	+	lmo0538	+	575365	575502	−	138(138)	0.475	16	0	13	.	.	lmo0537	2.5
3C	lmo0537	+	lmo0538	+	575671	575866	−	136(196)	0.478	13	0	13	.	.	lmo0537	2.5
3C	lmo0538	+	lmo0539	−	576528	577231	−	541(704)	0.487	65	0	13	.	−17.70	lmo0538	3
3C	lmo0538	+	lmo0539	−	577367	577631	−	226(265)	0.444	499	388	13	.	−22.60	lmo0538	4
Antisense transcripts of methylases																
3D	lmo0581	−	iap	−	617842	618101	+	164(260)	0.403	55	0	13	.	−21.20	lmo0581	3
3D	lmo0581	−	iap	−	618619	618728	+	110(110)	0.442	16	0	13	.	.	lmo0581	2.5
3E	lmo0934	+	lmo0935	+	971926	972112	−	140(187)	0.501	16	0	13	0.974	.	lmo0934	3
3E	lmo0935	+	lmo0936	+	972702	972980	−	221(279)	0.407	39	0	13	.	.	lmo0935/lmo0936	2.75
3E	lmo0936	+	lmo0937	−	973369	973508	−	91(140)	0.391	1750	222	13	0.975	.	lmo0936	3
3F	lmo0996	+	clpE	−	1026660	1026870	−	104(211)	0.400	400	12	13	.	−23.40	lmo0996	3.5
Antisense transcript of *kdpABCD* operon																
4	lmo2676	+	lmo2677	−	2748401	2748684	−	223(284)	0.380	25	42	13	0.990	−13.00	lmo2676	3
4	lmo2676	+	lmo2677	−	2748684	2754031	+	4647(5348)	0.429	228	35	13	0.973	−17.96	lmo2677/lmo2678/lmo2679/kdpC/kdpB	4.25

Selected asRNAs and their genomic location, syntenic genes (UpOrf, DownOrf), corresponding GC-content and length (in brackets extended lengths for asRNA detection). IC – number of reads mapped to this region from intracellular library; EC – number of reads mapped to this region from extracellular library; P – number of closely related *Listeria* serotypes, with a homologous region identified in a genome-wide multiple sequence alignment; RNAz – p-Value of *de novo* ncRNA prediction of RNAz; RNAL – MFE of locally stable secondary structures, calculated by RNALfold; Score – Score assigned in this study. The complete list of all novel ncRNA candidates can be viewed at the supplemental page.

Internalins (Inls) are a large group of proteins containing leucine-rich-repeats (LRR) and are known to play an important role in host-pathogen-interactions. The bacterial cell-surface anchored proteins InlA and InlB are required for cell-, tissue- and organ-specific invasion of *L. monocytogenes*. InlA engages the cell-junction protein E-Cadherin as its cellular receptor and InlB uses the hepatocyte growth factor receptor (HGFR, c-Met) for internalization [39]. Another cell-surface bound internalin is InlK, which binds to the Major Vault Protein (MVP) and thereby shields the bacterium from autophagy [40]. The secreted internalin InlC interacts directly with IKK*α*, a subunit of the I*κ*B kinase complex, which is critical for the phosphorylation of I*κ*B and activation of NF-*κ*B, to suppress the inflammatory response [41].

The regulation of internalins is relevant to understand the virulence of *L. monocytogenes*. Previous studies showed that the master virulence regulatory protein PrfA regulates several internalins, e.g., *inlAB* and *inlC* [42]. Moreover, transcriptional regulation by the alternative sigma factor SigB was reported for several internalins, e.g., *inlA*, *inlB*, *lmo0263* and *lmo0610* [43], [44].

Using RNA-seq, we showed in this study that internalins encoded by *lmo0333* (*inII*) and *lmo1136* are subject of antisense transcriptional regulation by long non-coding antisense RNAs (lasRNAs) las0333 and las1136. *Lmo1136* is presumed to encode an internalin [20] which has not been studied so far. InlI was recently described and investigated by Sabet *et al.* [45] in the mouse infection model, but a knockout mutant for the *inlI* gene did not exhibit any difference in virulence when compared to the wild type [45].

The long antisense transcripts of internalin have a length of 163 nt and 493 nt (Tab. 3). According to the expression levels those transcripts are presumably even longer, 1214 nt and 1617 nt respectively (see Fig. 3A,B). For *lmo0333* another antisense transcript of only 15 nt length, which is covered by 121 uniquely mapped reads, was detected. The number of reads mapping to the proposed lasRNAs varies between 28 and 121 reads. Interestingly transcription seems to be specific for *Listeria* grown in macrophages (intracellular) as for the extracellular condition no expression was observed.

We quantified the extra- and intracellular expression levels by qRT-PCR for all five selected lasRNAs (see Fig. 2A) and their corresponding mRNA transcripts (see Fig. 2B). All lasRNAs were up-regulated in the intracellular compartment. mRNA targets of *las0333* and *las0936* were repressed, whereas transcription of *lmo0996*, *lmo1136* and *lmo2677* was induced under intracellular conditions. This might indicate that these newly identified lasRNAs are involved in depression of target mRNAs (*lmo0333* and *lmo0936*) and stabilization of mRNA transcripts (*lmo0996*, *lmo1136* and *lmo2677*), what has been also reported for other lasRNA transcripts, e.g. from *Prochlorococcus* [46].

### Novel long antisense transcript (2,400 nt–3,800 nt)

An extremely long antisense transcript, spanning at least 2,400 nt (see Tab. 3), was observed antisense to *lmo0537* and *lmo0538*. Gene *lmo0537* codes for an amidohydrolase including a dimerization domain. The transcript contains four asRNA candidate loci, which might be also a single long antisense transcript. It is likely that the detected lasRNA influences its antisense genes *lmo0538* and *lmo0537*. However, this cannot be proven yet. Nevertheless, a rough inverse transcript pattern of the proteins and their expected antisense regulators is observable (see Fig. 3C). The antisense transcript of *lmo0537* seems to be specific for intracellular conditions.

### Antisense transcripts to methylases

Another example that caught our attention are antisense transcripts of various methylases, namely *lmo0581* (a predicted SAM-dependent methyltransferase, see Fig. 3D), *lmo0935* (CspR protein, a rRNA methylase homolog, see Fig. 3E) and *lmo0996* (similar to a methylated DNA protein cystein methyltransferase, see Fig. 3F).

The antisense transcript of *lmo0581* was mainly observed for the intracellular condition (see Fig. 3D). Even though the expression is very low in some parts, it is spanning *lmo0581* (1161 nt) completely. Gene *lmo0581* itself is transcribed under extracellular and intracellular growth conditions.

The second putative lasRNA spans three genes (see Fig. 3E): it was detected antisense to *lmo0936* (similar to nitroflavin-reductase), *lmo0935* (SpoU, rRNA methylase) and *lmo0934* (uncharacterized Fe-S protein, energy production and conversion). One striking feature of this candidate is its length of 2,500 nt. Even though the transcription rate is very low in some regions, an antisense transcript of this length is remarkable. Whereas the transcription of the lasRNA is specific for intracellular grown *Listeria*, the genes are covered with reads originating from both growth conditions.

The third methyltransferase having putative asRNA transcripts is *lmo0996* (see Fig. 3F), which is similar to methylated DNA-protein-cystein methyltransferase. This asRNA is an intergenic transcript and appears to be transcribed continuously with its syntenic genes *lmo0997* (*clpE*, ATP-dependent protease) and *lmo0995* (predicted acetyltransferase). The intergenic transcription is observed only in intracellularly grown *Listeria*. This indicates that the reads cannot be simply attributed to extended 5′ or 3′ UTRs, but are rather a putative specific intracellular ncRNA. We observed only very low transcription for the protein gene *lmo0996*, neither for extracellular nor for intracellular conditions.

All of the above mentioned antisense transcripts are short (91–221 nt) and covered by 16–1750 reads (see Tab. 3). The read pattern of the ncRNA candidates is rather unsteady. A direct influence of the lasRNAs to the methylases can be only hypothesized.

### The *kdpEDABC* operon is controlled by an extremely long non-coding antisense RNA

**Figure 4:**
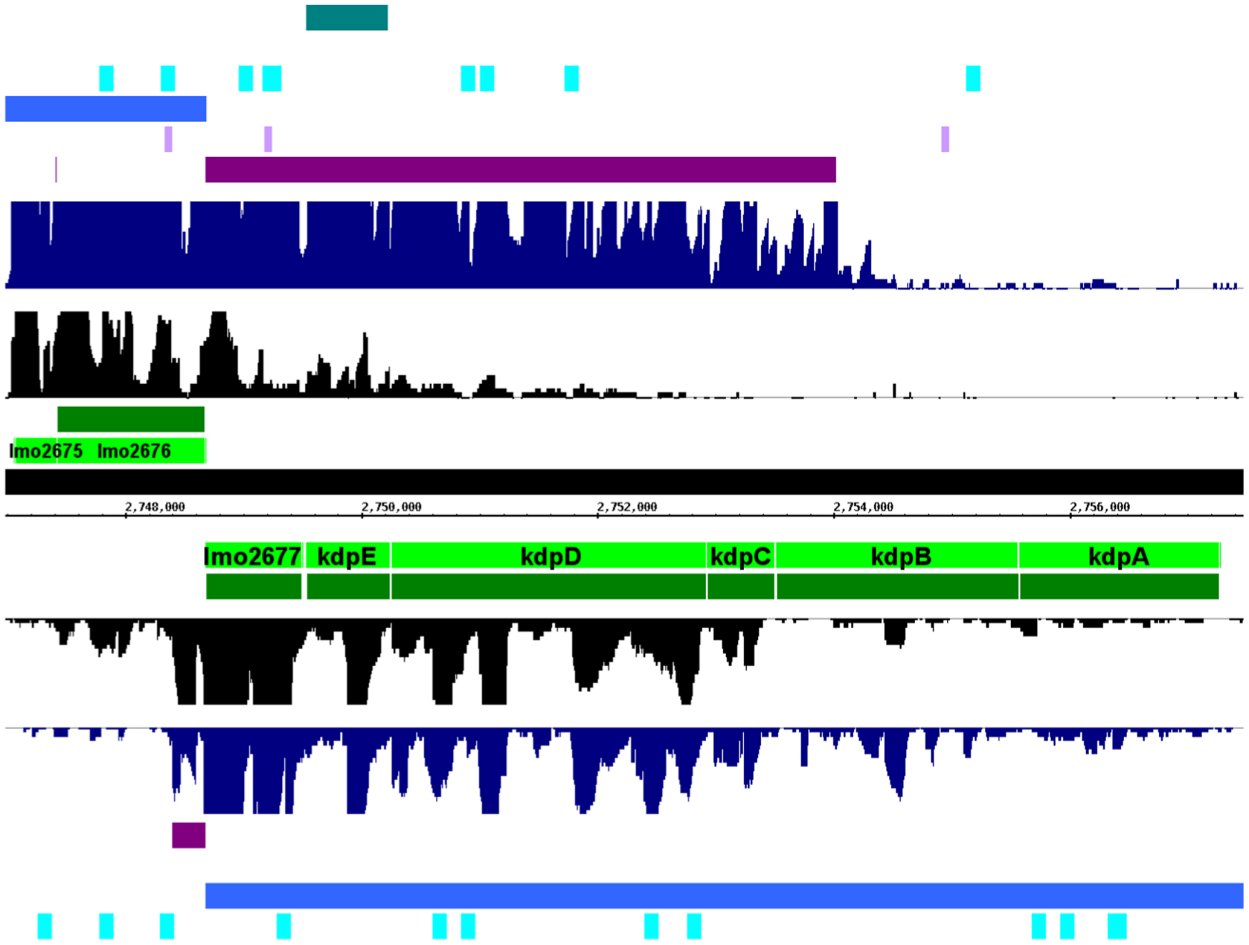
Transcription of a selected long asRNA (lasRNA): *kdpABCD* operon. Number of displayed reads is limited to 20. Dark purple – detected ncRNA candidates; lightgreen – NCBI annotation; darkgreen – BacProt annotation; black – reads of the extracellular library; dark blue – reads of the intracellular library; violet – locally stable secondary structure (analyzed with RNALfold); blue – conserved region among other *L. monocytogenes* serotypes (analyzed with POMAGO); cyan blue – potential new ncRNAs predicted by RNAz; pink – annotated ncRNAs; teal green – ncRNA candidates of previous studies.

Among the newly detected lasRNAs we have identified a very long antisense RNA of about 5,400 nt which completely covers the region from *lmo2677* up to *lmo2680* and partially the gene *kdpB* (see Tab. 3 and Fig. 4). This lasRNA is strongly activated during the intracellular growth phase of the pathogen and was confirmed by qRT-PCR (see Fig. 2) analysis. Previously Wurtzel *et al.* [15] described an asRNA for *lmo2678*, which is transcribed under exponential growth at 37°C and is controlled by SigB. The gene *lmo2678* encodes the response regulator (KdpE) of a two component system (TCS) together with a cognate histidine kinase (KdpD) encoded by *lmo2679* [47]. Under high-osmolarity conditions the KdpED TCS regulates the adjacent *kdpABC* operon which is responsible for high-affinity potassium uptake as previously reported for *Escherichia coli* [48]. Several different reports described KdpED to be involved in intracellular survival of pathogenic bacteria, for example *Staphylococcus aureus*, entero-haemorrhagic *E. coli*, *Salmonella typhimurium* and *Yersinia pestis* [49]. In *L. monocytogenes*, however, it does not seem to play an important role in virulence [50]. This is supported by the observation that the entire locus *lmo2677*–*lmo2681(kdpB)* is down-regulated by massive antisense transcription. This suggests that alternative uptake systems exist to ensure potassium uptake. Such systems have been already reported for *B. subtilis* [51]. It is, however, unclear why this long asRNAs is necessary to block the *kdpED* TCS and *kdpABC* operon under intracellular conditions. Why is a short asRNA, as described by Wurtzel *et al.* [15], produced during extracellular growth conditions, not sufficient to stop transcription of *lmo2678* and the *kdpED* TCS/*kdpABC* operon? We speculate that these asRNAs do not only stringently regulate transcription in *cis*, but also in *trans*.

Recently Mellin *et al.* [16] reported that in the presence of vitamin B_12_, the corresponding riboswitch induces transcriptional termination. This causes an antisense RNA *aspocR* to be transcribed as a short transcript. In the absence of vitamin B_12_, *aspocR* is transcribed as a long antisense RNA, inhibiting *pocR* expression [16]. A similar non-classical function could be also assumed for the *kdpEDABC* interfering las2677/las2678 RNAs.

Furthermore, there seems to be a correlation between the asRNA read pattern and the start and stop sites of the operon genes. For example, for *lmo2678/kdpE* there is an increase and decrease correlating with the start and stop positions of this (see Fig. 4). It is tempting to speculate whether this lasRNA is originating from *lmo2676* or not. In case it is originating from *lmo2676*, the transcript might resemble an excludon. Interestingly another ncRNA candidate was detected directly downstream to *lmo2677* (see Fig. 4). Nevertheless, this seems to be a separate transcript and not an extended 3′UTR, since there is an obvious decrease of reads at the end of *lmo2677*. This ∼300 nt RNA antisense to the 5′part of *lmo2676* is stronger expressed under extracellular conditions.

To confirm our newly identified asRNAs in another *L. monocytogenes* serotype 1/2a strain, we have preformed additional RNA-seq experiments (unpublished RNA-seq data, online supplementary material) with the commonly used *L. monocytogenes* strain 10403S grown under extra- and intracellular conditions. Comparison of presence/absence of the las0333, las0936, las0996, las1136 and las2677 showed a similar occurrence of these asRNAs between *L. monocytogenes* strain 10403S and EGD-e. This implicates a conserved expression mechanism for *L. monocytogenes* serotype 1/2a strains for these selected asRNA candidates.

In addition, we have also tested the transcription regulator mutant of *L. monocytogenes* EGD-e Δ*prfA* under the same experimental conditions described above. Our RNA-seq analysis (unpublished RNA-seq data, online supplementary material) showed that all above mentioned asRNAs were independently controlled by the master virulence regulator PrfA. Furthermore, these new RNA-seq data warrant detailed investigation in future.

## Conclusion

We systematically used the semiconductor sequencing technology for RNA-seq to identify ncRNAs and determine the difference of extra- and intracellular growth conditions. We reported bacterial antisense transcripts with a size up to 5,400 nt. It would be interesting to use our pipeline to examine whether similar transcripts can be observed in other bacteria. Further work has to be done to fully understand the functional role of these long non-coding antisense RNAs in bacterial physiology. Particularly in the case of the *kdpABCD* operon, the regulation of K^+^ by long non-coding antisense RNAs now deserves further attention.

## References

[1] Vazquez-Boland JA, Kuhn M, Berche P, Chakraborty T, Dominguez-Bernal G, et al (2001) *Listeria* pathogenesis and molecular virulence determinants. ClinMicrobiolRev 14: 584–640. 10.1128/CMR.14.3.584-640.2001 PMC88991 11432815

[2] Swaminathan B, Gerner-Smidt P (2007) The epidemiology of human listeriosis. MicrobesInfect 9: 1236–1243. 10.1016/j.micinf.2007.05.011 17720602

[3] Hain T, Chatterjee SS, Ghai R, Kuenne CT, Billion A, et al (2007) Pathogenomics of *Listeria* spp. IntJMedMicrobiol 297: 541–557. 10.1016/j.ijmm.2007.03.016 17482873

[4] Graves LM, Helsel LO, Steigerwalt AG, Morey RE, Daneshvar MI, et al (2010) *Listeria marthii* sp. nov., isolated from the natural environment, Finger Lakes National Forest. IntJSystEvolMicrobiol 60: 1280–1288. 10.1099/ijs.0.014118-0 19667380

[5] Leclercq A, Clermont D, Bizet C, Grimont PA, Le Fleche-Mateos A, et al (2010) *Listeria rocourtiae* sp. nov. IntJSystEvolMicrobiol 60: 2210–2214. 10.1099/ijs.0.017376-0 19915117

[6] Bertsch D, Rau J, Eugster MR, Haug MC, Lawson PA, et al (2013) *Listeria fleischmannii* sp. nov., isolated from cheese. IntJSystEvolMicrobiol 63: 526–532. 10.1099/ijs.0.036947-0 22523164

[7] den Bakker HC, Manuel CS, Fortes ED, Wiedmann M, Nightingale KK (2013) Genome sequencing identifies *Listeria fleischmannii* subsp. *coloradonensis* subsp. nov., isolated from a ranch. IntJSystEvolMicrobiol 63: 3257–3268. 10.1099/ijs.0.048587-0 23524352

[8] Lang HE, Neuhaus K, Scherer S (2013) *Listeria weihenstephanensis* sp. nov., isolated from the water plant *Lemna trisulca* taken from a freshwater pond. IntJSystEvolMicrobiol 63: 641–647. 10.1099/ijs.0.036830-0 22544790

[9] Kuenne C, Billion A, Mraheil MA, Strittmatter A, Daniel R, et al (2013) Reassessment of the *Listeria monocytogenes* pan-genome reveals dynamic integration hotspots and mobile genetic elements as major components of the accessory genome. BMCGenomics 14: 47. 10.1186/1471-2164-14-47 PMC3556495 23339658

[10] Hain T, Steinweg C, Kuenne CT, Billion A, Ghai R, et al (2006) Whole-genome sequence of *Listeria welshimeri* reveals common steps in genome reduction with *Listeria innocua* as compared to *Listeria monocytogenes* . JBacteriol 188: 7405–7415. 16936040 10.1128/JB.00758-06 PMC1636279

[11] Mraheil MA, Billion A, Mohamed W, Mukherjee K, Kuenne C, et al (2011) The intracellular sRNA transcriptome of *Listeria monocytogenes* during growth in macrophages. Nucleic Acids Res 39: 4235–4248. 21278422 10.1093/nar/gkr033 PMC3105390

[12] Hain T, Ghai R, Billion A, Kuenne CT, Steinweg C, et al (2012) Comparative genomics and transcriptomics of lineages I, II, and III strains of *Listeria monocytogenes* . BMCGenomics 13: 144. 10.1186/1471-2164-13-144 PMC3464598 22530965

[13] Toledo-Arana A, Dussurget O, Nikitas G, Sesto N, Guet-Revillet H, et al (2009) The *Listeria* transcriptional landscape from saprophytism to virulence. Nature 459: 950–956. 19448609 10.1038/nature08080

[14] Oliver HF, Orsi RH, Ponnala L, Keich U, Wang W, et al (2009) Deep RNA sequencing of *L. monocytogenes* reveals overlapping and extensive stationary phase and sigma B-dependent transcriptomes, including multiple highly transcribed noncoding RNAs. BMC Genomics 10: 641. 20042087 10.1186/1471-2164-10-641 PMC2813243

[15] Wurtzel O, Sesto N, Mellin JR, Karunker I, Edelheit S, et al (2012) Comparative transcriptomics of pathogenic and non-pathogenic *Listeria* species. Mol Syst Biol 8: 583. 22617957 10.1038/msb.2012.11 PMC3377988

[16] Mellin JR, Tiensuu T, Becavin C, Gouin E, Johansson J, et al (2013) A riboswitch-regulated antisense RNA in *Listeria monocytogenes* . ProcNatlAcadSciUSA 110: 13132–13137. 10.1073/pnas.1304795110 PMC3740843 23878253

[17] Raghavan R, Sloan DB, Ochman H (2012) Antisense transcription is pervasive but rarely conserved in enteric bacteria. MBio 3. 10.1128/mBio.00156-12 PMC3419515 22872780

[18] Nicolas P, Mader U, Dervyn E, Rochat T, Leduc A, et al (2012) Condition-dependent transcriptome reveals high-level regulatory architecture in *Bacillus subtilis* . Science 335: 1103–1106. 22383849 10.1126/science.1206848

[19] Arnvig KB, Comas I, Thomson NR, Houghton J, Boshoff HI, et al (2011) Sequence-based analysis uncovers an abundance of non-coding RNA in the total transcriptome of *Mycobacterium tuberculosis* . PLoSPathog 7: e1002342. 10.1371/journal.ppat.1002342 PMC3207917 22072964

[20] Glaser P, Frangeul L, Buchrieser C, Rusniok C, Amend A, et al (2001) Comparative genomics of *Listeria* species. Science 294: 849–852. 11679669 10.1126/science.1063447

[21] Bécavin C, Bouchier C, Lechat P, Archambaud C, Creno S, et al (2014) Comparison of widely used *Listeria monocytogenes* strains EGD, 10403S, and EGD-e highlights genomic variations underlying differences in pathogenicity. MBio 5: 00969–00914. 10.1128/mBio.00969-14 PMC3977354 24667708

[22] Chakraborty T, Leimeister-Wächter M, Domann E, Hartl M, Goebel W, et al (1992) Coordinate regulation of virulence genes in *Listeria monocytogenes* requires the product of the *prfA* gene. J Bacteriol 174: 568–574. 1729245 10.1128/jb.174.2.568-574.1992 PMC205751

[23] Chatterjee SS, Hossain H, Otten S, Kuenne C, Kuchmina K, et al (2006) Intracellular gene expression profile of *Listeria monocytogenes* . InfectImmun 74: 1323–1338. 10.1128/IAI.74.2.1323-1338.2006 PMC1360297 16428782

[24] Tasara T, Stephan R (2007) Evaluation of housekeeping genes in *Listeria monocytogenes* as potential internal control references for normalizing mRNA expression levels in stress adaptation models using real-time PCR. FEMS MicrobiolLett 269: 265–272. 10.1111/j.1574-6968.2007.00633.x 17263845

[25] Pfaffl MW (2001) A new mathematical model for relative quantification in real-time RT-PCR. Nucleic Acids Res 29: e45. 11328886 10.1093/nar/29.9.e45 PMC55695

[26] Hain T, Ghai R, Billion A, Kuenne CT, Steinweg C, et al (2012) Comparative genomics and transcriptomics of lineages I, II, and III strains of *Listeria monocytogenes* . BMC Genomics 13: 144. 22530965 10.1186/1471-2164-13-144 PMC3464598

[27] Lechner M, FindeißS, Marz M, Stadler P (2013) Bacprot: A protein annotation tool for bacteria. in progress.

[28] Lowe TM, Eddy SR (1997) tRNAscan-SE: a program for improved detection of transfer RNA genes in genomic sequence. Nucleic Acids Res 25: 955–964. 9023104 10.1093/nar/25.5.955 PMC146525

[29] Lagesen K, Hallin P, Rodland EA, Staerfeldt HH, Rognes T, et al (2007) RNAmmer: consistent and rapid annotation of ribosomal RNA genes. Nucleic Acids Res 35: 3100–3108. 17452365 10.1093/nar/gkm160 PMC1888812

[30] Altschul SF, Gish W, Miller W, Myers EW, Lipman DJ (1990) Basic local alignment search tool. J Mol Biol 215: 403–410. 2231712 10.1016/S0022-2836(05)80360-2

[31] Nawrocki EP, Kolbe DL, Eddy SR (2009) Infernal 1.0: inference of RNA alignments. Bioinformatics 25: 1335–1337. 19307242 10.1093/bioinformatics/btp157 PMC2732312

[32] Gardner PP, Daub J, Tate JG, Nawrocki EP, Kolbe DL, et al (2009) Rfam: updates to the RNA families database. Nucleic Acids Res 37: D136–D140. 18953034 10.1093/nar/gkn766 PMC2686503

[33] Wieseke N, Lechner M, Ludwig M, Marz M (2013) POMAGO: Multiple Genome-Wide Alignment Tool for Bacteria. In: Bioinformatics Research and Applications. Springer, number 1 in Lecture Notes in Computer Science, pp. pp 249–260.

[34] Washietl S, Hofacker IL, Stadler PF (2005) Fast and reliable prediction of noncoding RNAs. Proc Natl Acad Sci U S A 102: 2454–2459. 15665081 10.1073/pnas.0409169102 PMC548974

[35] Hofacker IL, Priwitzer B, Stadler PF (2004) Prediction of locally stable RNA secondary structures for genome-wide surveys. Bioinformatics 20: 186–190. 14734309 10.1093/bioinformatics/btg388

[36] Hoffmann S, Otto C, Kurtz S, Sharma CM, Khaitovich P, et al (2009) Fast mapping of short sequences with mismatches, insertions and deletions using index structures. PLoS Comput Biol 5: e1000502. 19750212 10.1371/journal.pcbi.1000502 PMC2730575

[37] Griffiths-Jones S, Bateman A, Marshall M, Khanna A, Eddy SR (2003) Rfam: an RNA family database. Nucleic Acids Res 31: 439–441. 12520045 10.1093/nar/gkg006 PMC165453

[38] Christiansen JK, Nielsen JS, Ebersbach T, Valentin-Hansen P, Sogaard-Andersen L, et al (2006) Identification of small Hfq-binding RNAs in *Listeria monocytogenes* . RNA 12: 1383–1396. 16682563 10.1261/rna.49706 PMC1484441

[39] Pizarro-Cerda J, Kuhbacher A, Cossart P (2012) Entry of *Listeria monocytogenes* in mammalian epithelial cells: an updated view. Cold Spring HarbPerspectMed 2.. 10.1101/cshperspect.a010009 PMC3543101 23125201

[40] Dortet L, Mostowy S, Samba-Louaka A, Gouin E, Nahori MA, et al (2011) Recruitment of the major vault protein by InlK: a *Listeria monocytogenes* strategy to avoid autophagy. PLoSPathog 7: e1002168. 10.1371/journal.ppat.1002168 PMC3150275 21829365

[41] Gouin E, Adib-Conquy M, Balestrino D, Nahori MA, Villiers V, et al (2010) The *Listeria monocytogenes* InlC protein interferes with innate immune responses by targeting the i*κ*B kinase subunit IKK*α* . ProcNatlAcadSciUSA 107: 17333–17338. 10.1073/pnas.1007765107 PMC2951401 20855622

[42] de las HA, Cain RJ, Bielecka MK, Vazquez-Boland JA (2011) Regulation of *Listeria* virulence: Prfa master and commander. CurrOpinMicrobiol 14: 118–127. 10.1016/j.mib.2011.01.005 21388862

[43] Hain T, Hossain H, Chatterjee SS, Machata S, Volk U, et al (2008) Temporal transcriptomic analysis of the *Listeria monocytogenes* EGD-e sigmaB regulon. BMCMicrobiol 8: 20. 10.1186/1471-2180-8-20 PMC2248587 18226246

[44] Kazmierczak MJ, Mithoe SC, Boor KJ, Wiedmann M (2003) *Listeria monocytogenes* sigma B regulates stress response and virulence functions. JBacteriol 185: 5722–5734. 13129943 10.1128/JB.185.19.5722-5734.2003 PMC193959

[45] Sabet C, Lecuit M, Cabanes D, Cossart P, Bierne H (2005) LPXTG protein InlJ, a newly identified internalin involved in *Listeria monocytogenes* virulence. InfectImmun 73: 6912–6922. 10.1128/IAI.73.10.6912-6922.2005 PMC1230919 16177371

[46] Stazic D, Lindell D, Steglich C (2011) Antisense RNA protects mRNA from RNase E degradation by RNA-RNA duplex formation during phage infection. Nucleic Acids Res 39: 4890–4899. 21325266 10.1093/nar/gkr037 PMC3113571

[47] Brondsted L, Kallipolitis BH, Ingmer H, Knochel S (2003) kdpE and a putative RsbQ homologue contribute to growth of *Listeria monocytogenes* at high osmolarity and low temperature. FEMS MicrobiolLett 219: 233–239. 10.1016/S0378-1097(03)00052-1 12620626

[48] Polarek JW, Williams G, Epstein W (1992) The products of the *kdpDE* operon are required for expression of the Kdp ATPase of *Escherichia coli* . JBacteriol 174: 2145–2151. 1532387 10.1128/jb.174.7.2145-2151.1992 PMC205832

[49] Freeman ZN, Dorus S, Waterfield NR (2013) The KdpD/KdpE two-component system: integrating K^+^ homeostasis and virulence. PLoSPathog 9: e1003201. 10.1371/journal.ppat.1003201 PMC3610689 23555240

[50] Williams T, Bauer S, Beier D, Kuhn M (2005) Construction and characterization of *Listeria monocytogenes* mutants with in-frame deletions in the response regulator genes identified in the genome sequence. InfectImmun 73: 3152–3159. 10.1128/IAI.73.5.3152-3159.2005 PMC1087338 15845524

[51] Holtmann G, Bakker EP, Uozumi N, Bremer E (2003) KtrAB and KtrCD: two k^+^ uptake systems in *Bacillus subtilis* and their role in adaptation to hypertonicity. JBacteriol 185: 1289–1298. 12562800 10.1128/JB.185.4.1289-1298.2003 PMC142857

